# Collaboration in the formulation and implementation of policies for noncommunicable diseases in South Africa

**DOI:** 10.4102/hsag.v28i0.2100

**Published:** 2023-03-20

**Authors:** Richard M. Rasesemola

**Affiliations:** 1 Department of Nursing, Faculty of Health Sciences, University of Johannesburg, Johannesburg, South Africa

**Keywords:** multisectoral collaboration, multisectoral engagement, coordinating committee, policy improvement strategies, prevention and control of non-communicable diseases

## Abstract

**Background:**

Collaboration between health and other sectors is necessary and much needed when addressing health issues. The health sector alone does not possess all the necessary resources to address health problems in the country. Thus, the burden of disease because of the noncommunicable diseases (NCDs) requires interventions that are sometimes beyond the health sector’s mandate.

**Aim:**

To investigate collaboration in the policy formulation process for prevention and control of NCDs in South Africa. This article presents strategies that could aid South African government to ensure collaboration by various sectors in addressing the NCDs.

**Setting:**

This study took place in the provincial Department of Health (DoH) of seven South African provinces.

**Methods:**

This was quantitative descriptive study done among purposefully sampled respondents from various health portfolios from seven provincial Departments of Health. Data were collected using questionnaires and analysed using descriptive statistical data analysis techniques.

**Results:**

The results indicated that the DoH collaborates with private and government stakeholders in the policy formulation and implementation process but excludes them in the setting the health agenda, adoption of policy options and policy evaluation.

**Conclusion:**

The lack of participation by other stakeholders in the critical phases of policy formulation will result in continued burden of disease because of poor prevention and control of NCDs in the country.

**Contribution:**

This article provides recommendations that would ensure collaboration among various sectors to accelerate the response to the prevention and control of NCDs in South Africa.

## Introduction

There is an enormous and a growing global burden of disease because of noncommunicable diseases (NCD) (World Health Organization [WHO] 2022:Online). Over 63% (36 million) of global deaths in 2008 were linked to NCDs (Agide & Shakibazadeh 2018:356), the deaths increased to 70% (39.2 million) by 2015 with over three quarters of such deaths occurring in low- and middle-income countries (NCD Countdown 2030 Collaborators 2018). These deaths are expected to increase by up to 75% by 2030 around the world, with the greatest increase expected to be in the sub-Saharan African region (Gouda et al. 2019:e1375). Noncommunicable diseases made up 50% of all the illnesses that caused deaths in South Africa in 2010, and by 2015 the deaths from NCDs had surpassed deaths from communicable diseases at 55.5% (Department of Statistics 2017:27). By 2020, over 50% of deaths in South Africa were attributed to NCDs (WHO 2020:171). Noncommunicable diseases would likely continue to be the leading cause of death in the coming years unless action is taken (eds. Massyn et al. 2015:195).

The risk factors for the NCDs are linked to the complex interaction of multiple factors such as the socioeconomic and political context, environmental, social and cultural factors, material and lifestyle factors known as social determinants of health (Marmot & Bell 2019:10). Interventions to address some of these risk factors are beyond health sector’s mandate and require action from the non-health sectors. So, the prevention and control of NCDs require coherence among various private and government stakeholders because the health system alone does not have all the necessary tools to solve all these challenges (Kickbusch & Gleicher 2012:viii). Therefore, collaboration among various stakeholders from government and private sector is essential. The involvement of multiple stakeholders from different sectors is essential for sharing necessary resources and fostering a sense of ownership in designing and implementing programmes to ensure effective prevention and control of NCDs (Juma et al. 2019:21).

Multisectoral collaboration and cooperation at different levels of governance were adopted by the South African signatories at the World Health Assembly as a commitment to increase efforts to address the burden of NCDs in the country (Spires et al. 2016:36). The government, through the Department of Health (DoH), developed policies aimed to address NCDs that included framework for the establishment of coordinating structures that would be responsible to ensure implementation of such policy plans (DoH 2015:Online). The process requires the *National Health Act No. 61 of 2003* to make provisions for the establishment of such coordinating structures by the DoH. However, in its current form, the *National Health Act No. 61 of 2003* does not make provisions for the national coordinating committee to provide oversight into the implementation of plans and activities for the prevention and control of NCDs beyond the health sector. The implications include poor collaboration with non-health stakeholders from both government and private sector. Other implications include passive resistance by food and beverage manufacturing industries and advertising industries without any form of reprisal by the government.

The South African government developed several strategies, policies and programmes to address the prevention and control of NCD, such as the *Strategy for the Prevention and Control of Obesity in South Africa* 2015–2020 (DoH 2015), the *National Cancer Strategic Framework for South Africa* 2017–2022 (DoH 2017) and *National Mental Health Policy Framework and Strategic Plan* 2013–2020 (DoH 2013). Despite strategies, policies and programmes, little has been achieved in terms of reducing the burden of diseases related to these conditions. Therefore, the aim of this article is to present collaboration in the formulation and implementation of the policies for the prevention and control of NCDs and present policy improvement strategies that could aid in addressing the prevention and control of NCDs in South Africa.

## Research methods and design

### Study design

The study was quantitative descriptive research done among 57 purposefully sampled respondents from seven provinces including Limpopo, Western Cape, Northern Cape, KwaZulu-Natal, Eastern Cape, Northwest and Free State in South Africa. Quantitative research design allowed the researcher to collect data without being in contact with the respondent using a questionnaire. Descriptive research involved the collection of quantitative information that produced data that was tabulated along a continuum in numerical form (Gray, Grove & Sutherland 2017:23; Polit & Beck 2017:741). Descriptive research focused on accurate pictures related to the formulation and implementation of policies for the prevention and control of NCDs as reported by the respondents. Data collected produced results that were depicted using graphs and tables to assist the researcher to thoroughly outline and describe the collaboration between various stakeholders in different phases of policy formulation.

### Research setting

The research setting in this study includes the provincial DoH of Limpopo, Western Cape, Northern Cape, KwaZulu-Natal, Eastern Cape, North West and Free State. Provincial DoH is a critical entry point in strengthening of health systems and attaining some of the international and national declarations on NCDs. It was crucial for this research study to include respondents from the provincial level of health governance in South Africa because it is where the implementation of the national DoH functions is ensured, strategic direction and objectives are set and decisions relating to the allocation of resources to meet the strategic goals and objectives including the prevention and control of NCDs are made (*National Health Act No. 61 of 2003*).

### Study population and sampling strategy

The population included the provincial public authorities from various health portfolios responsible for making health policies and executing health policies in South Africa. The purposive sampling method was used to identify respondents, and it is based on the judgement of the researcher regarding respondents who are especially knowledgeable about the research being conducted (Brink, Van der Walt & Van Rensburg 2018:126). Based on the accessible population size of 72 respondents, the sample size was calculated using Slovin’s formula; thus, the minimum sample size for this study was 20 respondents at 5% margin of error. However, the sample size was 57 respondents.

### Data collection

Respondents were invited via telephone and email. Respondents were sent an information leaflet and consent form to sign if they agreed to participate in the study. Questionnaires were sent to the respondent via email, in person via hand delivery and some respondents opted to complete the questionnaire via telephonic interview. Telephonic interview as a data collection tool involved orally asking the respondents quantitative questions. The process included the researcher asking the questions to the respondents as they are reflected in the questionnaire and writing responses down verbatim on behalf of the respondents. The advantage of telephonic interview in a quantitative research study is that it allows the researcher flexibility and access that would usually be unavailable through traditional methods.

The questionnaire was most useful because it is one of the most practical ways and cost-efficient way to quickly collect massive amounts of information from many people in a short period. Furthermore, questionnaire as a data collection tool was decided upon because the respondents feel a great sense of anonymity and are more likely to provide honest answers in their responses (Brink et al. 2018:147). Because of the coronavirus disease 2019 (COVID-19) national lockdown restrictions that allowed minimal travelling between the provinces and physical meetings, the researcher was able to collect relevant data from respondents at a distance and from those who were unable to meet physically by using questionnaires that were sent via various methods.

Questionnaires were sent to 57 potential respondents who indicated their willingness to participate and who have signed consent forms and responses were obtained from 57 respondents via email, hand-completed questionnaire or telephonic interview across all seven provinces. Data analysis began immediately after the completion of data collection process.

### Data analysis

Data analysis was performed to include descriptive statistics, which refers to procedures that describe numerical data, and they assist in organising, summarising and interpreting quantitative data (Bhandari 2020:Online). Data management and descriptive analysis procedures were done using Microsoft 365 Excel Spreadsheet Software, and results were presented in tabular and graphical formats.

### Ethical considerations

The protection of human subjects through the application of appropriate ethical principles is important in any research study (Arifin 2018:30). Fundamental research ethics principles serve as a framework for conducting research; therefore, the research was approved by the Research Ethics Committee of the Tshwane University of Technology (FCRE 2020/01/007 [FCPS O1] [SCI]). Ethical clearance from Provincial DoH was also obtained (Free State, Limpopo, North West, Western Cape, KwaZulu-Natal, Eastern Cape & Northern Cape).

The right to self-determination and autonomy was ensured. The right to self-determination was ensured through obtaining an informed consent, where the respondents voluntarily confirm their willingness to participate in a research study, after being informed of all aspects of the research relevant to their decision to participate (Yip, Han & Sng 2016:695). Autonomy, also known as respect for people, demands that competent respondents make their own decisions, be recognised and respected by preventing the imposition of unwanted decisions (Owonikoko 2013:234). According to Vilma (2018:22), full autonomy requires research respondents to understand what they are being asked to do, make a reasoned judgement about the effect participation would have on them and decide to participate free from coercion or influence. To ensure the ethical principle of autonomy, respondents received an information leaflet because the researcher was required to furnish the potential research respondents with full disclosure about the nature of the study, its risks, benefits and alternatives.

Principles of justice and fairness include respondents’ right to fair treatment and privacy (Varkey 2020:18). The right to fair treatment means that the selection of study respondents is based on the research requirements rather than their vulnerability or compromised position (Varkey 2020:18). To ensure justice in this study, the researcher treated the respondents fairly and equally, and there was no victimisation of those respondents who refused to participate in the study. Privacy was ensured by protecting respondents’ identities and any information that could link them with their responses.

## Results

### Sample demographic characteristics

Table 1 presents the characteristics of the sample included in the study.

**TABLE 1:** Sample demographic characteristics.

Position	Frequency (*n*)	Percentage (%)
Director	31	54.5
Deputy Director	6	10.5
Deputy Director General	6	10.5
Assistant Director	2	3.5
Assistant Deputy Director	12	21.0

**Total**	**57**	**100**

As reflected in Table 1, the sample included 57 respondents comprising Directors, Deputy Directors, Deputy Director General, Assistant Directors and Assistant Deputy Directors from various portfolios in the provincial DoH of seven South African provinces.

### Collaboration in the policy formulation process

Respondents reported on collaboration between the DoH and other government and non-government stakeholders in the policymaking process. The policymaking process entails how health problems are identified and included in the government’s priorities or health policy agenda is set. Furthermore, policymaking process entails how solutions are formulated, and the best solutions to address the identified problems are identified and adopted. Lastly, policymaking process entails how such solutions are put into action and an assessment of the objectives of the chosen solutions against the achievements of such solutions as indicated by the respondents.

**FIGURE 1:**
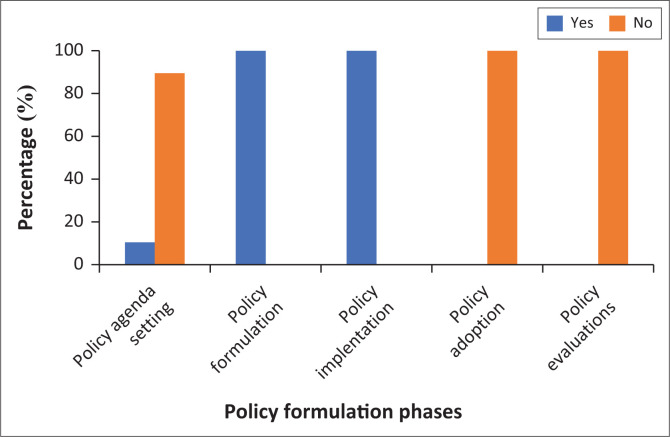
Collaboration for policy formulation process.

The results relating to the collaboration with stakeholders in the policy formation process are reflected in Figure 1.

All the respondents indicated that there is collaboration with stakeholders during health policy formulation and implementation. Only 10.5% (*n* = 6) of respondents indicated that there is collaboration with stakeholders for the health policy agenda setting. None of the respondents indicated collaboration with stakeholders during health policy adoption as well as policy evaluation.

**FIGURE 2:**
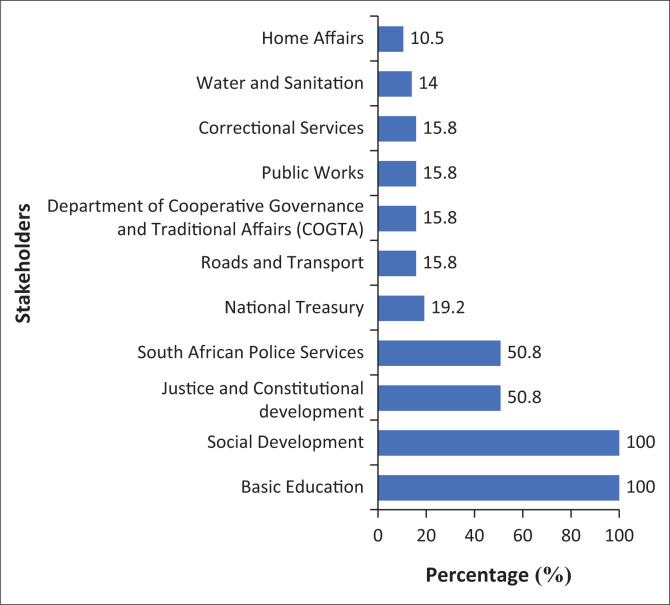
Government stakeholders collaborating in policy formulation process.

**FIGURE 3:**
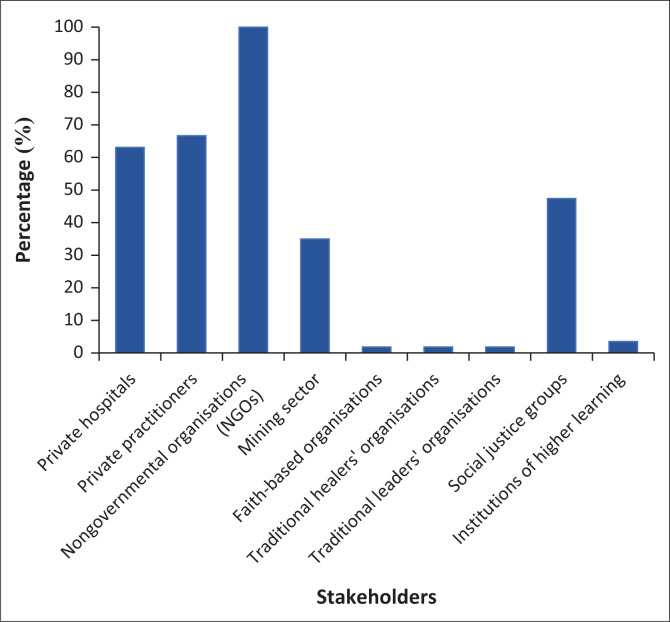
Private stakeholders for policy formulation process.

The respondents reported that there is collaboration by the DoH with both government and non-government stakeholders as reflected in Figure 2 and Figure 3, respectively.

All the respondents indicated that there is collaboration with the Department of Basic Education and the Department of Social Development as stakeholders at the provincial level. More than a half (50.8%, *n* = 29) of the respondents indicated collaboration with the South African Police Services and the Department of Justice and Constitutional Development as some of their important stakeholders in their respective provinces. Only 14% (*n* = 8) respondents indicated collaboration with the Department of Water and Sanitation, the Department of Roads and Transport and National Department of Public Works as some of the important stakeholders as reflected in Figure 2.

The private stakeholders that were involved in the policy formulation process at the provincial level as reported by the respondents at provincial health are reflected in Figure 3.

All the respondents reported that they collaborate with nongovernmental organisations (NGOs) in policy formulation process at the provincial level. Faith-based organisations, traditional healers’ and traditional leaders organisations were inadequately incorporated in the healthcare matters as indicated by only 1.8% (*n* = 1) of the respondents reporting that these organisations form part of the stakeholders in policymaking process at the provincial level. Institutions of higher learning were also insufficiently incorporated at 3.5% (*n* = 2). All respondents indicated that they collaborate with NGOs in the policy formulation process, followed by private practitioners, private hospitals, mining sector and social justice groups at 66.7% (*n* = 38), 63.1% (*n* = 35), 35% (*n* = 30) and 47.4% (*n* = 27), respectively.

## Validity and reliability

Content validity, which is used to assess whether the instrument adequately covers all the content that it should with respect to the variables under investigation, was ensured (Heale & Twycross 2015:66). Content validity was ensured by means of face validity where the researcher designed the data collection instruments after continuous consultation with NCDs and policy analysis experts to ensure that the instrument measured the intended concepts.

Reliability refers to the consistency and dependability of a research instrument measuring a variable (Polit & Beck 2017:764). Data reliability can be ensured using internal consistency (Bless, Higson-Smith & Sithole 2013:226). To promote internal consistency, the researcher should ensure that an instrument meant to measure the phenomenon under investigation measures the phenomenon and yields approximately similar responses each time it is completed by the same subjects (Heale & Twycross 2015:67). To ensure internal consistency in this study, the data collection instruments were designed based on Stages Heuristic, a known policy analysis framework.

## Discussion

Policymaking and implementation process can be long processes that include a series of activities to ensure that health problems of public concern are addressed. Frameworks such as Stages Heuristics break down the complex policymaking and implementation process into separate and manageable stages to assist policy researchers to explore and comprehend the complex process of policymaking and implementation (Gültekin 2014:48; Public Health Ontario 2016:2). This framework focuses on how problems are defined, agendas are set, policies are formulated, decisions are made and policies are implemented and evaluated (eds. Gilson, Agyepong & Shiffman 2018:10).

Stages heuristics further promotes an understanding of various stakeholders who influence, decide on and implement various stages of the policy process (Cattaneo 2018:11). Therefore, this research assessed collaboration in the policy formulation and implementation process of the policies for prevention and control of NCDs in South Africa. The results indicated that the DoH collaborated with various stakeholders such as various government departments and private stakeholders in the formulation process of policies for the prevention and control of cancers, obesity and mental and behavioural disorders in South Africa. There was a lack of collaboration with the stakeholders during the agenda setting and policy adoption in the policy formulation process. The lack of collaboration during agenda setting means that there were no external stakeholders involved when health problems were identified and included in the government’s priorities. The lack of collaboration between the DoH and other stakeholders during policy adoption means that the DoH acts alone when it comes to making the choice for the best solutions to address the identified health problems. This research further reported on the lack of collaboration with stakeholders during policy evaluation, which entails an assessment of the objectives of the chosen solutions against the achievements of such solutions.

Collaboration involves different stakeholders working together in a coherent manner to achieve a shared goal (Juma et al. 2019:2). The lack of collaboration in some of the important aspects of policy formulation and implementation process occurs despite some reassuring evidence from the literature that suggests that intersectoral collaboration is feasible and has the potential to fast-track the prevention and control of NCDs. Literature indicates that policy plans are more likely to be implemented effectively if they are developed and implemented in collaboration with a full range of partners who can significantly contribute to implementation from both within and outside the health sector (WHO 2013:33). Therefore, to ensure successful implementation of policies for the prevention and control of NCDs, Del Busto and colleagues (2019:9) posit that collaboration is significant and that governments must develop collaborative work between various stakeholders at different levels, both from within and outside the health sector.

Internationally, collaboration across various sectors and levels of governance is a key intervention strategy in the prevention and control NCDs, and it is a major component of a whole-of-government approach (Juma et al. 2019:3). Marmot and Bell (2019:10) also state the importance of aligning priorities across various sectors and stakeholders to support consistent approach to the formulation and implementation of policies for the prevention and control of NCDs. The WHO also supports collaboration across government departments, civil society and private sector to accelerate response towards the prevention and control of NCDs (WHO 2013:33, 2020). These stakeholders should be involved as partners of the governments in advocating, planning, shaping, implementing, monitoring and evaluating health policies (WHO 2013:11).

The lack of collaboration in some aspects of policymaking and implementation as reflected in this study might be because of lack of initiative from the DoH to play a pivotal role in mobilising stakeholders and creating a platform for collaboration. Another reason for a lack of collaboration might be because other non-health government departments might be lacking the interest to collaborate with the DoH in policymaking and implementation. This perception is similar to that of Juma and colleagues (2019:4), who stated that the departments of Finance and Trade and Industry might not support certain policy stance such as imposing advertising bans and excise duties on alcohol and tobacco because they might be concerned about the loss of revenue from taxing alcohol consumers and about job losses. Furthermore, Ndinda and Hongoro (2017:48) also reported that alcohol and advertising industries declined to support further restrictions on alcohol bans proposed by the DoH for the prevention of NCDs citing consequences such as job losses and slumped economic growth.

Therefore, to effectively address NCDs in South Africa, it is crucial that the government recognises that health is a common objective and achieving it requires coherence among various stakeholders. This is because health system alone does not have the tools to solve all health challenges (Kickbusch & Gleicher 2012:viii). Such as the lack of basic sporting facilities necessary to promote physical activity and healthy lifestyle in South African schools (Amnesty International 2020:37), which is beyond the DoH mandate. However, the lack of physical activity facilities in schools might lead to the development of obesity and related NCDs. Therefore, other sectors such as the Department of Basic Education and Infrastructure Development must be involved in addressing these challenges to ensure the provision of sporting facilities to allow physical activity among school children, necessary to prevent obesity and related NCDs.

Other challenges include poor road conditions that do not allow people to have access to the opportunities for physical activities and active transportation in the rural areas where the majority of rural and poor communities reside (Nyawo & Mashau 2019:12553). The consequences of poor road infrastructures and the resulting poor physical activity could lead to the development of various NCDs; however, the provision of roads is not the mandate of the health system. Therefore, collaboration between the DoH and other stakeholders is essential and very much needed. The involvement of multiple sectors is essential for fostering a sense of ownership among the parties involved in designing and implementing different aspects of health programmes (Juma et al. 2019:2).

To facilitate collaboration between the DoH, various government departments and private sector, the author recommends the establishment of mechanisms for facilitating collaborative efforts among these stakeholders by the DoH. These mechanisms entail the establishment of multisectoral national coordinating committee and the legislation of multisectoral collaboration for the prevention and control of NCDs in South Africa. In its current form, the *National Health Act No. 61 of 2003* does not make provision for the establishment of coordinating committee that goes beyond the health sector to ensure collaboration and implementation of policy plans for the prevention and control of NCDs in South Africa. Therefore, the *National Health Act No. 61 of 2003* should be amended to make provisions for the DoH to establish the national multisectoral coordinating committee constituting representatives from various levels of governance, across all the provinces, with representatives from various government and non-government stakeholders with necessary leadership and governance skills. Such national multisectoral coordinating committee should be equipped with the regulatory and enforcement powers through the *National Health Act No. 61 of 2003* to regulate compliance to the NCD prevention and control efforts by the health and non-health sectors from both government and non-government stakeholders.

The national multisectoral coordinating committee would further promote the establishment of centralised policy-coordinating mechanisms, coordinate the mobilisation and equitable distribution of necessary resources for the prevention and control of NCDs in the country. Through its regulatory powers, the multisectoral coordinating committee would also provide oversight into the implementation of plans and activities for the prevention and control of NCDs beyond the health sector and would also ensure that each stakeholder accounts for their role in the prevention and control on NCDs through the establishment of surveillance and reporting mechanisms.

The author envisages that establishing the multisectoral national coordinating committee for the prevention and control of NCDs would ensure equitable distribution of resources necessary for the prevention and control of NCDs across the country and at various levels of healthcare. This is important in the South African context because of the differences in the health outcomes across various regions in the country, with the poorest regions suffering the most because of shortage of various types of resources necessary for the prevention and control of NCDs. This multisectoral national coordinating committee would provide leadership on the implementation plans necessary to ensure positive gains towards addressing NCDs and to ensure that the relevant stakeholders play an active role in achieving the progress.

In the Philippines, the national multisectoral governance and coordinating structures that provide oversight into NCD policy engagement beyond the health sector have successfully facilitated multisectoral action for the development and implementation of policies for the prevention and control of NCDs (Rasanathan et al. 2017:4). Similarly in the United Arab Emirates, the Ministry of Health yielded positive gains towards the prevention and control of NCDs by establishing of national multisectoral committee that ensures that each necessary sector accounts for implementation of plans aimed at addressing the NCDs (Fadhil, Bin Belaila & Razzak 2019:7). Thus, the establishment of multisectoral national coordinating committee in South Africa would also facilitate joint plans that outline shared inter-departmental goals with integrated budgets and resources to ensure coordinated and effective response towards the prevention and control of NCDs.

## Conclusion

The results indicated that the DoH collaborates with various private and governmental stakeholders for the development of policies for the prevention and control of NCDs in South Africa. Such stakeholders are not fully incorporated in the policy formulation process. Because some determinants of NCDs are beyond health system’s mandate, addressing them requires inputs from non-health stakeholders. Thus, the exclusion of non-health stakeholders in the critical phases of policy formulation has serious implications. Such implications include poor prevention and control of NCDs in the country and the resulting burden of disease and possible resistance by the stakeholders that should be part of the NCDs policy formulation and implementation. Therefore, to ensure that health is not just a common objective but also a common responsibility that requires coherence across sectors, the author suggested policy improvement strategies that include the establishment of multisectoral national coordinating committee and the legislation of multisectoral engagement to improve the prevention and control of NCDs in South Africa.
